# Invisible mother-daughter heredity

**DOI:** 10.1093/eurpub/ckac131.451

**Published:** 2022-10-25

**Authors:** E Capitani, C Lorenzini, E Baragatti, L Alaimo, G Messina, N Nante

**Affiliations:** 1 Post Graduate School of Public Health, University of Siena, Siena, Italy; 2 Degree Course in Obstetrics, University of Siena, Siena, Italy; 3 Department of Molecular and Developmental Medicine, University of Siena, Siena, Italy

## Abstract

**Background:**

The type of maternal caregiving impacts children’s growth and can lead to heritable changes in gene expression. When women become mothers, they adopt parents’ behaviours similar to those received in the family of origin. The study investigates if a birth’s social and cultural content can be seen as heredity transmitted from mother to daughter.

**Materials and methods:**

A retrospective study was conducted on a group of mothers recruited across Italy through the social network Facebook. The study was carried out using a questionnaire administered from July to September 2021. The survey consisted of 21 questions. The analysis was carried out using STATA 14.

**Results:**

Our sample consisted of 6051 mothers; the mean age was 37.7 years. Womens born by spontaneous birth has 2.1 times higher risk of having a spontaneous birth for their first child. Those born by operative labour have 2.7 times higher risk of having an operative delivery for their first child. Finally, those born by caesarean section are 2.3 times more likely to have a caesarean section for their first child. On the other hand, those born by preterm labour have 1.8 times higher risk of delivering their first child preterm. Those who were breastfed have 2 times higher risk of breastfeeding their first child. Women who have been told their birth is an extraordinary event are 2 times more likely to consider the birth of their first child as a problematic but still extraordinary event. Those who received a description of childbirth from their mother as a problematic event are twice as likely to consider the birth of their first child as a traumatic event overall.

**Conclusions:**

The results show that transmission, written in the psyche, in preverbal and in internalisations derived from the relationship with one’s mother, is true and strongly present. Also, the ways of one’s own birth are so strong as to have repercussions on the daughter’s own and subsequently also on her children.

**Key messages:**

• The unconscious objects, which are projected onto the children, can also take the form of both physical and psychic somatisations, which are repeated cyclically between generations.

• The way in which birth is cared for and the quality of care provided at this unique time in a woman’s life will leave an imprint not only on the woman herself, but also on future generations.

